# Emergency department utilization among family caregivers of persons living with dementia

**DOI:** 10.1002/alz70860_102170

**Published:** 2025-12-23

**Authors:** Cameron Gettel, Pei‐Chun Cha, Craig Rothenberg, Anita Chary, Justine Seidenfeld, Ula Hwang, Terri Fried, Joan K. Monin, Arjun Venkatesh

**Affiliations:** ^1^ Yale School of Medicine, NEW HAVEN, CT, USA; ^2^ Baylor College of Medicine, Houston, TX, USA; ^3^ Durham VA Healthcare System, Durham, NC, USA; ^4^ NYU Langone Health, New York, NY, USA; ^5^ Yale School of Medicine, New Haven, CT, USA; ^6^ Yale School of Public Health, New Haven, CT, USA

## Abstract

**Background:**

Caring for a person living with dementia (PLWD) can place significant psychological and physical strain on family caregivers, making caregiver burden a critical concern amid the increasing global prevalence of dementia. While many studies have focused on resources to support caregiving or care‐seeking patterns of PLWD, few have examined caregivers' own healthcare utilization. We sought to characterize emergency department (ED) visit use among family caregivers of PLWD.

**Methods:**

We performed an observational cohort study using the 2016 to March 2020 Medical Expenditure Panel Survey data. We identified PLWD based on ICD‐10 codes and caregivers through specified relationships to the reference PLWD. The primary outcome was the rate of monthly ED visits among PLWD family caregivers (cases) compared to matched MEPS participants not caring for PLWD family members (controls), using a 1:1 propensity score matching approach to adjust for caregiver age, gender, race, education, and health conditions. We determined risk ratios between groups to account for differences in follow‐up time and quantify differences in ED visit rates.

**Results:**

The analytic sample included 510 participants, comprising 255 cases and 255 controls – 54.7% were female, 49.8% were White, and the average age was 45.5 years old. Aggregated across all study years, PLWD caregivers had 4.57 ED visits/100 person‐months, while matched controls had 3.51 ED visits/100 person‐months, representing a 30% higher rate of ED visits for PLWD caregivers (95% CI: 1.02, 1.66) compared to controls.

**Conclusion:**

Caregivers of PLWD have higher ED visit rates than matched controls not caring for family members with dementia. These findings highlight the potential healthcare challenges faced by dementia caregivers and emphasize the need for targeted interventions and policies to support their health and well‐being.

